# Preparation and mechanical characterization of pressed carbonized wood sawdust bio-composite

**DOI:** 10.1038/s41598-025-98658-w

**Published:** 2025-04-29

**Authors:** Hossein Rahmani, Augonis Algirdas, Anhelina Shestavetska, Danute Vaiciukyniene

**Affiliations:** Faculty of Civil Engineering and Architecture, Kaunas University of Technology, Kaunas, Lithuania

**Keywords:** Bio-composite, Wood sawdust, Shale ash, Concrete blocks, Eco-friendly materials, Composites, Mechanical properties

## Abstract

This study investigates the development and performance of carbonized bio-composites derived from wood sawdust, integrated with sustainable binders such as cement, lime, and shale ash, to create environmentally friendly construction materials. The research systematically optimizes mix compositions and curing techniques to enhance mechanical properties, softening, and sustainability, with a focus on reducing cement content and mitigating CO_2_ emissions. Wood sawdust, treated with various solutions (water, Al_2_(SO_4_)_3_, CaCl_2_, Ca(OH)_2_), was combined with binders and additives (sand, shale ash) in precise proportions, followed by a controlled carbonization process (19% CO_2_, 65% RH). Compressive strength tests revealed that cement-based composites with water-treated sawdust and 20% sand achieved a 44% strength increase (up to 9.6 MPa), while 30% cement replacement with shale ash yielded a 55% strength gain and improved water resistance (softening coefficient: 0.55). Carbonization, preceded by air-drying, further enhanced strength by 12% and density by 2%, demonstrating superior durability under moisture exposure. X-ray diffraction (XRD) and scanning electron microscopy (SEM) analyses confirmed the formation of calcite and a cohesive microstructure, respectively, underpinning the mechanical improvements. CO_2_ emissions were reduced by up to 65% in optimized formulations compared to traditional cement production, aligning with circular economy principles. These bio-composites, suitable for lightweight masonry applications, outperform prior sawdust-based materials (1–3 MPa) in strength and sustainability. This work advances the field of sustainable construction by offering a scalable, high-performance alternative to conventional materials, with rigorous methodology and robust data supporting its potential for industrial adoption.

## Introduction

The infrastructure of the whole world relies heavily on cement, which is a fundamental component of contemporary building materials^1,2^. Additionally, concrete, which is mostly made up of cement, is the substance that is used the most by people all over the world, with water being the only material that is consumed more than concrete^3–5^. It is estimated that around 626 kg of cement are produced by each individual on the planet^6^. The global production of cement has increased at an exponential rate, surpassing 4 billion tons yearly^7^. This huge scale shows the material’s environmental and energy-intensive issues while also highlighting the material’s indispensability with regard to the environment^8,9^. The International Energy Agency estimates that the cement sector is responsible for almost seven percent of the world’s CO_2_ emissions, making it a substantial contributor to the phenomenon of climate change^10,11^. Therefore, the need to discover new and environmentally friendly alternatives to cement manufacturing has become even more pressing^12,13^.

It is the disposal of wood waste that presents the environmental challenge that the timber business must tackle^14,15^. There are issues associated with recycling and reuse of wood leftovers since they are often treated with hazardous chemicals^16^. There is a further escalation of environmental challenges in Europe due to the fact that less than fifty percent of wood waste gets recycled^17,18^. Wood-cement composites and other innovative materials may provide potential solutions to this issue^19,20^. The utilization of wood waste in cement formulations as a substitute for ash or aggregate, or as a filler, presents significant potential for the development of innovative building materials that are sustainable, and environmentally friendly^21,22^. Composite materials, including wood-cement boards, offer advantages in various structural and soundproofing applications, attributed to their excellent acoustic insulation properties and environmental sustainability^23,24^.

Recent studies have investigated the intricate interactions among wood extractives, wood particles, and cement matrices, revealing important insights into the mechanisms that affect performance and compatibility^25,26^. The investigations have identified essential factors, including the chemical composition of wood extractives, their capacity to disrupt cement hydration, and methods to alleviate these impacts to improve material stability and strength^27^. The advancements in utilizing wood waste in construction materials highlight the feasibility of this approach^26^. However, they also reveal notable knowledge gaps, especially regarding the long-term behavior, structural integrity, and durability of wood-cement composites when subjected to various environmental conditions, including moisture exposure, temperature fluctuations, and mechanical stresses^27–29^.

Studies explore sustainable construction materials using alternative binders like marble dust^30^, wheat starch-based NaCl-binder^31^, and biochar with CO₂ curing^32^ for clay stabilization. Fiber reinforcement, including glass^33^ and carbon fiber^34^, enhances material performance, while Fahmi et al. studied starchy NaCl-binder strength^35^. Hanafi and Ekinci emphasized local materials^36^, such as alluvium clay with Portland cement and coal bottom ash^37^ or triple-binder marine clay^38^. Mollaei et al.^39^ and Hanafi et al.^40^ explored geopolymer waste and microstructure. Studies also cover sand void ratio^41^, basalt fiber-bottom ash composites^42^, geosynthetic reinforcement in sandy soils^43^, and wood-ash stabilized marine clay^44^. These studies point to the potential of local and waste materials for durable and low-cost construction.

In order to overcome these inadequacies, researchers are gradually embracing innovative alternatives such as bio-composites, which are manufactured via the use of contemporary pressing technology^45^. Using this technique, composite materials with superior environmental and structural features are created by combining scrap wood with a variety of binders and sustainable additives, such as shale ash^46,47^. The mechanical properties of these bio-composites are equal to those of traditional construction materials, such as strength and durability, but, at the same time, they significantly reduce the amount of conventional cement that is required^48^. Furthermore, the integration of sustainable additives not only solves the environmental issues that are involved with the manufacture of cement, but it also makes use of waste materials that would otherwise contribute to the overflow of landfills^49^. When it comes to the development of environmentally friendly construction technologies, bio-composites are a feasible option because of the combination of benefits that include resource efficiency and environmental sustainability^50^.

This study explores the potential of compressed bio-composites made from wood sawdust combined with binding agents cement, lime, and shale ash as sustainable alternatives for construction applications, with a focus on masonry. The primary aim is to develop eco-friendly materials that minimize environmental impact while maintaining structural integrity. Specific objectives include: (a) Reducing cement content by integrating sustainable additives like shale ash and sand, (b) Optimizing mixing and carbonization processes to enhance mechanical and physical properties and (c) Assessing the feasibility of these bio-composites for practical building applications. The research systematically investigates compressive strength, density, water resistance, and microstructural properties, using advanced analytical techniques such as SEM and XRD. By leveraging locally sourced wood waste from local area and incorporating shale ash, this study seeks to address the environmental challenges of cement production, which accounts for 7% of global CO_2_ emissions, and wood waste disposal, where less than 50% is recycled in Europe. The novelty lies in its holistic approach combining waste valorization, cement reduction, and carbonization optimization to produce ready-to-use masonry blocks that meet international sustainability standards. This work aligns with global efforts to transition the construction industry toward a circular economy, offering innovative, durable, and low-emission material solutions. This effort uses present developments in materials science to help the creation of creative cement substitutes and fits within the bigger context of global sustainability issues. The findings show possibility for lowering the environmental effect of building and supporting the industry’s circular economy change. This work has as its main tasks the following:

1. Utilizing sustainable materials such as shale ash and wood residue, bio-composites are being developed by minimizing the use of cement.
2. Utilizing environmentally favorable construction materials to mitigate CO_2_ emissions and dispose of wood residue.
3. Assessing the composites’ softening, physical properties, and mechanical strength.
4. Complying with sustainability objectives by minimizing the use of non-renewable materials and repurposing industrial residues.

## Materials and methods

This study focuses on the preparation and production of bio-composite materials using wood sawdust and various chemical solutions. Wood sawdust from Lithuania was chosen for its cleanliness and lack of contaminants. Four chemical solutions were created by dissolving specific substances in distilled water. These solutions were labeled as Solution A (tap water), Solution B (water mixed with Al_2_(SO_4_)_3_), Solution C (water combined with CaCl_2_), and Solution D (water integrated with Ca(OH)_2_). The wood sawdust was soaked in these solutions to encourage chemical interactions. After the soaking period, the wood sawdust was extracted from each solution, drained, and partially dried to achieve a moisture content of 30%. The conditioned wood sawdust was then mixed with a binder using a mechanical mixer or manual stirring rod until a uniform consistency was obtained. This ensured the integration of the sawdust throughout the binder matrix, creating a cohesive composite material for bio-composites production. To create bio-composite Specimens, cement was mixed with moist wood sawdust. The resulting composite material was shaped using a mixer and press machine, followed by a 28-day air-drying period to ensure dimensional stability. Different compositions were explored, including variations with sand. These Specimens were then air-dried and carbonized in a controlled chamber before undergoing subsequent analysis. Specimen preparation included dividing the mixed bio-composite material into molds and compacting it using a specialized pressing apparatus or hydraulic press. The compaction process was carefully calibrated to achieve the desired density and structural integrity of the Specimens. Following the molding process, the bio-composite Specimens were cured in a controlled environment, such as a curing chamber, with regulated exposure to air, water, and carbonization processes. The Specimens were then transferred to another controlled setting for consistent temperature and humidity maintenance.

### Initial materials

**Fig. 1:**
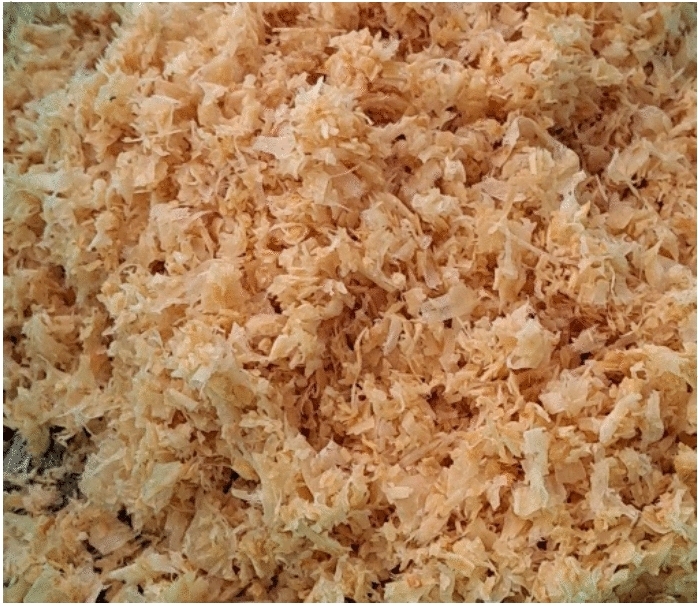
Dry wood sawdust used for composite Specimen preparation.

Wood waste: Wood sawdust obtained from a trusted supplier located in Lithuania, underscoring the necessity of securing material that is clean and free from contaminants (Fig. 1). This sawdust is waste material from Lithuanian wood industry.

**Fig. 2:**
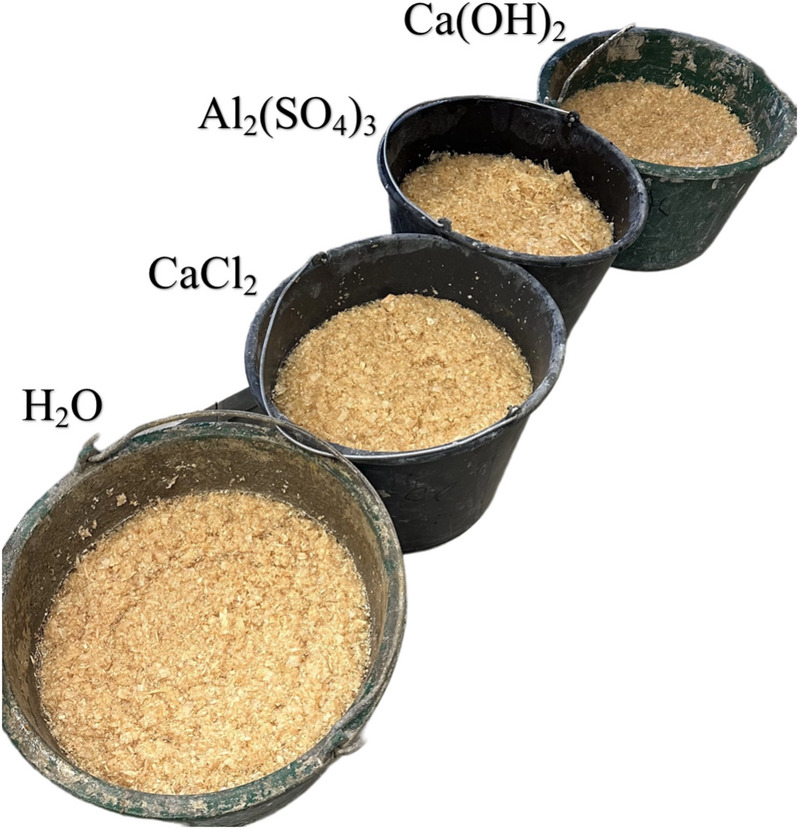
Wood sawdust after 7 days of soaking treatment in different solutions.

The choice of these kinds of materials for present study was because of their availability in local area. Following a detailed assessment, wood sawdust and cement were identified as the most suitable materials for the exploration of bio-composites. The wood sawdust were subjected to a rigorous process for sugar extraction, which involved soaking in four different chemical solution. The chemical treatment parameters included water (H_2_O), aqueous solutions of aluminium sulphate (Al_2_(SO_4_)_3_), calcium chloride (CaCl_2_), and calcium hydroxide (Ca(OH)_2_) (Fig. 2). This treatment of sawdust is recommended because it removes sugars that have acted as a harmful retardant to OPC hydration^51^.

Chemical solutions: Four unique solutions were prepared by dissolving the salts in distilled water (2.75%). These solutions were labeled as follows: Solution A (tap water), Solution B (water mixed with 0.22 kg/8 L, Al_2_(SO_4_)_3_), Solution C (water combined with 0.22 kg CaCl_2_), and Solution D (water integrated with 0.22 kg Ca(OH)_2_). To address wood suwdust treatment, precisely 0.8 kg of wood sawdust per 8 L (0.1 kg/l) was introduced into designated containers containing specific treatment solutions. Subsequently, the wood sawdust underwent soaking in these solutions for a 7 days to duration to facilitate chemical interactions.

Portland Cement: Based on the studies and investigations conducted, Portland cement type 1 with a compressive strength class of 42.5 MPa (CEM 1 42.5 R) in accordance with EN 197-1:2011 standard was used to achieve the desired mechanical strength and rapid setting. This cement was utilized in all processes and mixing designs present in this research.

Lime Powder: Based on the studies and investigations conducted, industrial powdered hydrated building calcium lime with 90% purity in accordance with EN 459-1 standard was used to achieve the desired mechanical strength and rapid setting. This lime was utilized in all processes and mixing designs present in this research.

Shale Ash: The shale ash used in the present study exhibits low self-cementing properties, which are influenced by the fineness and mineral composition of the ash. Its addition increases the porosity and enhances the insulating properties of bio-composites. Additionally, rapid carbonation is a key characteristic of the shale ash used in this study, indicating promising compressive strength and proposing it as a sustainable and waste-free solution.

#### Mixing process of bio-composites

Upon completion of the soaking period, the wood sawdust underwent a meticulous extraction process from each distinct chemical solution. This was followed by a carefully controlled drainage phase, complemented by a partial drying stage to achieve an optimal moisture content of 30%. Subsequently, the conditioned wood sawdust was methodically relocated to designated mixing vessels.

In these vessels, the sawdust and binder components were amalgamated with precision, utilizing either a mechanical mixer for efficiency or a manual stirring rod for finer control. The mixing process was continued until a uniform consistency was attained. This critical step ensured that the wood sawdust was seamlessly integrated throughout the binder matrix, forming a cohesive and evenly blended composite material, ready for subsequent processing stages in the creation of bio-composites.

#### Mix compositions for initial materials

The analysis detailed in Table 1 presents a comprehensive examination of wet wood sawdust, highlighting a notable water content that was quantified to be around 30%. This figure was meticulously calculated by comparing the mass of the sawdust in its initial, moisture-laden state against its mass post the drying phase. The process involved first recording the wet mass of the sawdust sample, which was then exposed to a systematic dehydration method. The drying was conducted in an oven, the temperature of which was carefully maintained at 70° C. The duration of this drying process was set to span 24 h, ensuring that all moisture was effectively eliminated.

**Table 1:** Compositon of initial materials for bio-composite curing conditions.

Type	NO.^1^	WWS^2^ (kg/m^3^)	PC^3^ (kg/m^3^)	LP^4^ (kg/m^3^)	S^5^ (kg/m^3^)	SA^6^ (kg/m^3^)	Solution	Curing Condition			W/S^10^ (Ratio)
								TAC^7^ (Day)	TCC^8^ (Day)	TH^9^ (Day)	
Bio-WWSPC											
A	6	358.50	1050.00	0.00	0.00	0.00	H_2_O	28	–	–	0.83
B	6	358.50	1050.00	0.00	0.00	0.00	Al_2_(SO_4_)_3_	28	–	–	0.83
C	6	358.50	1050.00	0.00	0.00	0.00	Ca(OH)_2_	28	–	–	0.83
D	6	358.50	1050.00	0.00	0.00	0.00	CaCl_2_	28	–	–	0.83
Bio-WWSLP											
A	6	358.50	0.00	478.00	0.00	0.00	H_2_O	28	–	–	0.13
B	6	358.50	0.00	478.00	0.00	0.00	Al_2_(SO_4_)_3_	28	–	–	0.13
C	6	358.50	0.00	478.00	0.00	0.00	Ca(OH)_2_	28	–	–	0.13
D	6	358.50	0.00	478.00	0.00	0.00	CaCl_2_	28	–	–	0.13
Bio-WWSPCS											
A-10*	6	358.50	1050.00	0.00	135.00	0.00	H_2_O	28	–	–	0.6960
B-20	6	358.50	1050.00	0.00	270.00	0.00	H_2_O	28	–	–	0.6960
Bio-WWSPCSSA											
A-30^#^	6	358.50	733.00	0.00	280.70	314.30	H_2_O	28	–	–	0.64
B-60	6	358.50	366.50	0.00	464.00	497.5	H_2_O	28	–	–	0.64
Bio-WWSPCSSA-C^+^											
A-30	6	358.50	733.00	0.00	280.70	314.30	H_2_O	28	0	0	0.64
B-30	6	358.50	733.00	0.00	280.70	314.30	H_2_O	0	2	26	0.64
C-30	6	358.50	733.00	0.00	280.70	314.30	H_2_O	1	2	25	0.64

^1^Number of specimens, ^2^Wet Wood Sawdust, ^3^Portland Cement, ^4^Lime Powder, ^5^Sand, ^6^SA, ^7^Time of air curing: before carbonization, ^8^Time of carbonization curing, ^9^Time of hardening: in air, ^10^Water/Solids.

*: A-10: 10% Sand (Relates to specimens with an additional 10% sand by weight.)

#: A-30: 30% Shale Ash (Relates to specimens with an replacement 30% shale ash to cement by weight.)

+ : Carbonization Curing: Represents specimens exposed to a 19% CO2 and 65% relative humidity (RH) environment for two days.

This rigorous approach to measuring the water content is crucial for understanding the material’s behavior in various applications, particularly in construction and manufacturing industries where the mechanical properties of materials like wood sawdust are integral to product quality and structural integrity.

Table 1 presents a comprehensive overview of the initial material compositions used to create various bio-composite Specimens for curing condition experiments. The table is divided into several sections based on different mix designs, each labeled with a Type identifier (e.g., Bio-WWSPC, Bio-WWSLP, Bio-WWSPCS, Bio-WWSPCSSA, and Bio-WWSPCSSA-C). Each section details the quantities of key components Wet Wood Sawdust (WWS), Portland Cement (PC), Lime Powder (LP), Sand (S), and Shale Ash (SA) measured in kilograms per cubic meter (kg/m^3^), along with the specific Chemical Solution used for treatment. The table also includes parameters related to the curing process, such as the Time of Air Curing (TAC) before carbonization, the Time of Carbonization Curing (TCC) using a controlled CO_2_ environment and the Time of Hardening in air (TH) along with the Water-to-Solids Ratio (W/S). For the Bio-WWSPC series, four variations (A-D) are explored, each utilizing a different chemical solution for sawdust treatment including, water (H_2_O), aluminum sulfate (Al_2_(SO_4_)_3_), calcium hydroxide (Ca(OH)_2_), and calcium chloride (CaCl_2_). The same format is applied to the Bio-WWSLP series where, lime is the binder while chemical solutions are used to treat wood sawdust. The Bio-WWSPCS series introduces two sand contents of 10% and 20%. The Bio-WWSPCSSA series explores partial cement replacement with shale ash, at 30% and 60%. Lastly, the Bio-WWSPCSSA-C series investigates different combinations of air-drying, carbonization chamber, and ambient temperature curing condition, in specimens with partial cement replacement with shale ash. The table highlights the precise methodology of each mix variation, including the use of a carbonization chamber (19% CO_2_ concentration and 65% relative humidity) for two days, offering a clear depiction of the experimental approach for creating different bio-composite Specimens for further study of their mechanical and physical properties.

The determined water content percentage significantly influenced subsequent stages of the experiment. Specifically, it revealed that approximately one-third of wet wood sawdust consists of water. This insight guided the experimental protocol, where intentionally excluding additional water was crucial for specimen creation. Consequently, the decision to omit extra water was prudent, recognizing that a substantial portion of wet wood sawdust inherently contains moisture, obviating the need for additional hydration in the experimental specimens. To calculate the dry wood sawdust amount (251 kg/m^3^), subtract the water content (108 kg/m^3^) from the total wet wood sawdust mass.

Expanding our research scope, we introduced innovative compositions that combined cement, wet wood sawdust, and sand in varying proportions. Table 1 provides details on two additional compositions analyzed for this purpose. Composition types Bio-WWSPC and Bio-WWSPCS included 1050 kg/m^3^ of cement, 359 kg/m^3^ of wet wood sawdust, and 135 kg/m^3^ of 0.4 mm sand. In contrast, Composition types Bio-WWSPCS (A10 and A20) maintained the same cement and sawdust levels but incorporated a higher sand content at 270 kg/m^3^. We meticulously crafted these new formulations into bio-composite Specimens using the same rigorous methodology as before. After a critical 28-day air-drying period, the resulting bio-composite Specimens were then prepared for carbonization or further testing, consistent with the established procedures in this study.

#### Bio-composite production and drying

After completing the preparation phase, we meticulously initiated the production of bio-composite Specimens. Each Speciment underwent careful craftsmanship, combining cement with wet wood sawdust. The resulting composite material was expertly shaped using a mixer and press machine, followed by a controlled 28-day air-drying process. This extended drying period was crucial to ensure the material’s dimensional stability. Building upon the earlier methodology involving cement, we meticulously crafted additional bio-composite Specimens. These new Specimens consisted of 478 kg/m^3^ of lime and 359 kg/m^3^ of wet wood sawdust (composition type Bio-WWSLP in Table 1). To ensure material stability, these composites also underwent an air-drying phase. Our dual approach to Specimen preparation underscores the methodical rigor in pursuit of consistent and reliable experimental outcomes.

Expanding our research scope, we introduced innovative compositions that combined cement, wet wood sawdust, and sand in varying proportions. Table 1 provides details on two additional compositions analyzed for this purpose. Composition types Bio-WWSPC and Bio-WWSPCS included 1050 kg/m^3^ of cement, 359 kg/m^3^ of wet wood sawdust, and 135 kg/m^3^ of 0.4 mm sand. In contrast, Composition types Bio-WWSPCS (A10 and A20) maintained the same cement and sawdust levels but incorporated a higher sand content at 270 kg/m^3^. We meticulously crafted these new formulations into bio-composite Specimens using the same rigorous methodology as before. After a critical 28-day air-drying period, the bio-composite Specimens entered a controlled chamber for carbonization, following established procedures from previous phases. The carbonization process involved two sequential stages: first, exposure to a controlled environment with 19% CO_2_ concentration, 65% relative humidity, and a stable 20°C temperature for three days. This was followed by a 25-day air-drying phase in an ambient environment, completing the carbonization process and preparing the Specimens for subsequent analysis.

#### Sample preparation

**Fig. 3:**
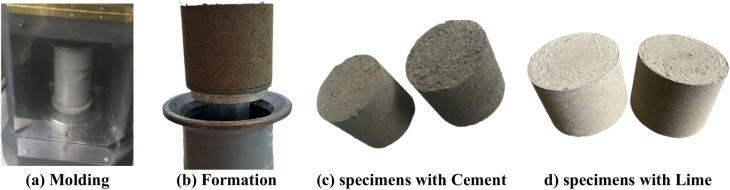
Bio-composite specimen preparation.

In the intricate process of preparing bio-composite materials, the division and compaction stages play pivotal roles, as depicted in Fig. 3. Initially, the mixed bio-composite material resulting from various chemical treatments is carefully divided. This material is then placed into individual sample molds, ensuring consistent filling to maintain uniformity across all samples. Subsequently, the compaction process begins. During this crucial phase, a specialized pressing apparatus or hydraulic press is used to compact the bio-composite mixture within the molds. The compaction is performed under a carefully calibrated pressure of 1.5 bars, executed through a series of 80 cycles. These specific pressure and duration settings are not arbitrary; they result from extensive preliminary testing aimed at determining optimal conditions for achieving the desired density and structural integrity in the bio-composite samples. Meticulous execution of these processes is essential to ensure that the final bio-composite materials meet the rigorous standards required for further testing and application.

#### Curing and conditioning

Following the precise molding process, the bio-composite Specimens were carefully reshaped to meet exact specifications and then placed in a controlled environment, such as a curing chamber. This chamber is meticulously designed to provide the ideal conditions for curing, including regulated exposure to air, water, and carbonization processes. The curing phase was conducted over a period deemed appropriate for the material’s composition, with temperatures set in accordance with the established norms for cement-based materials. This step is critical as it significantly influences the material’s final properties. Once the curing process was complete, the Specimens were transferred to another controlled setting, where temperature and humidity levels were maintained consistently. This environment was essential for the Specimens to reach an equilibrium moisture content, a state necessary for accurate testing and evaluation. The careful control of these conditions ensures the reliability and validity of the test results, ultimately reflecting the material’s suitability for real-world applications.

### Testing and mechanical analysis

After the bio-composite Specimens achieved the necessary conditioning, they underwent a series of tests to determine their physical and mechanical properties, adhering to standards such as EN12390-3^52^. These tests, crucial for assessing structural integrity and load-bearing capacity, included density measurement and compressive strength testing. The latter was conducted using a controlled cracking process, with a pressing ratio set at 100 N/S, following a 28-day drying period. Additionally, to mimic moisture exposure, four Specimens from different chemical treatments were submerged in water, a method that simulates real-world environmental conditions. The comprehensive data collected from these evaluations allowed for a detailed comparison of bio-composites treated with various solutions. This analysis was key to understanding each type’s performance, especially in water-cured scenarios, and was essential in determining the most suitable bio-composite formulations for specific construction applications. Microstructural and mineral composition analysis were conducted using XRD and SEM analysis. X-ray diffraction (XRD) patterns were collected for selected Specimens to assess their mineral composition and crystallinity. Scaning Electron Microscopy (SEM) was used to produce high-resolution images of the Specimens to assess their morphology and microstructure. Insights from this process are critical for customizing bio-composite materials to achieve optimal performance in designated uses, contributing to the advancement of more sustainable and efficient construction methodologies.

## Results and discussion

This research delves into the properties of carbonized bio-composites derived from wood sawdust. The study specifically focuses on the influence of cement and lime, sand content, shale ash as substitution, and curing methods on the mechanical strength of these specimens, as well as the potential to enhance their properties through carbonization. Furthermore, the investigation explores the microstructural characteristics of these composites and assesses the environmental implications of their production, including CO_2_ emissions. By comprehending these factors, our the aim is to contribute to the development of sustainable and high-performance bio-composite materials.

### Compressive strength

The present study delves into the evaluation of compressive strength in fabricated bio-composites. The influence of different solutions, the quantity of aggregate added, the replacement of cement with shale ash, and the effects of carbonization and curing processes are explored.

#### Effects of chemical solutions, sand content, and shale ash replacement

**Fig. 4:**
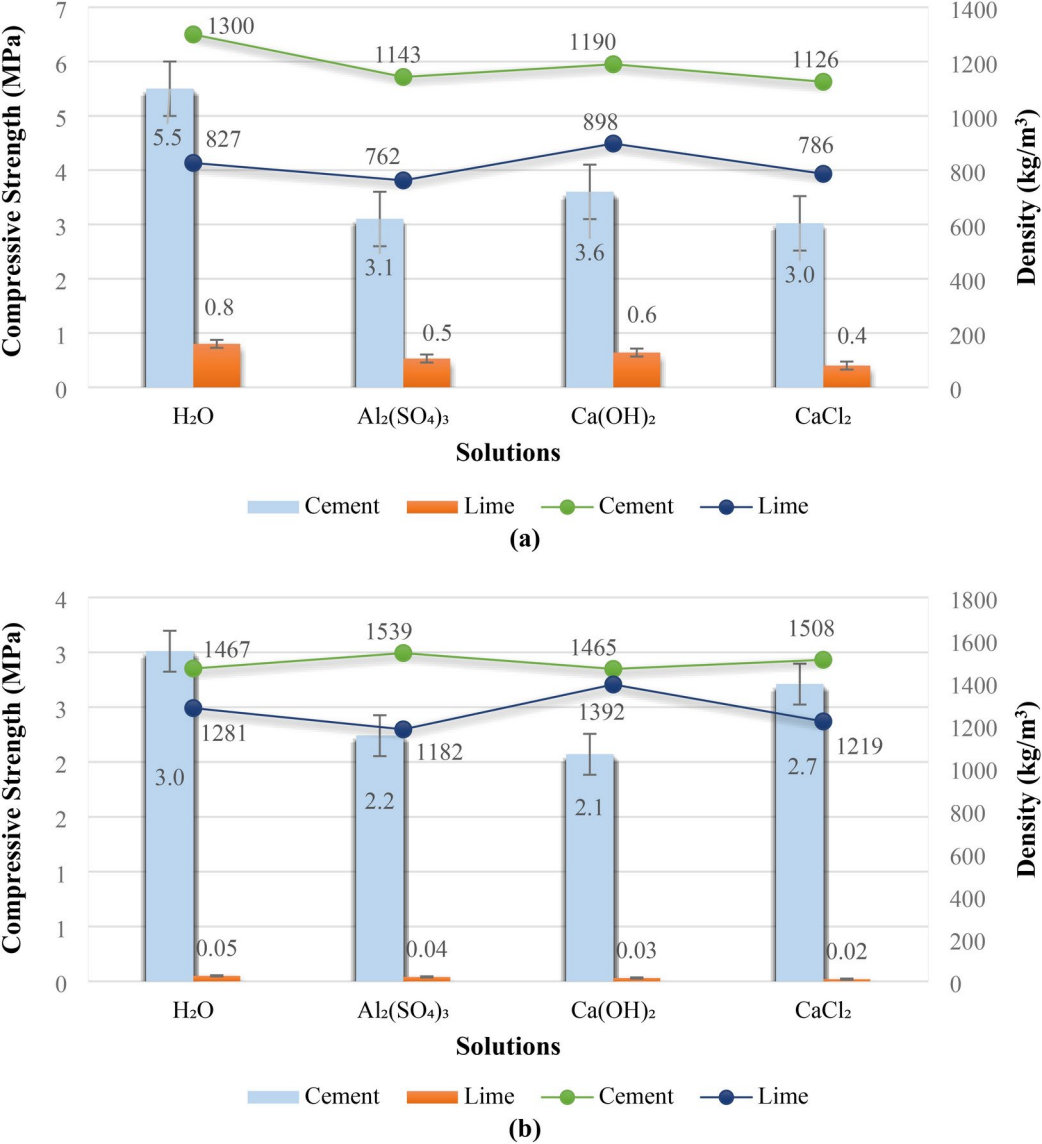
Compressive strength and density of cement and lime bio-composites: (**a**) before water exposure and (**b**) after water exposure.

With the aim of achieving an optimal mix design that balances mechanical properties and environmental sustainability, this section will explore the effects of incorporating chemical solutions, sand, and fly ash into the bio-composite mixture. Specifically, the focus will be on the impact of these additives on compressive strength, density, and water resistance, while also considering the potential to reduce CO_2_ emissions by partially replacing cement with fly ash. The results of compressive strength and density tests before and after water immersion are presented in Fig. 4. Morevere, Fig. 4a presents the compressive strength and density of the bio-composite Specimens before water immersion. It’s evident that the Specimens using cement as a binder achieved higher compressive strengths than the lime-based Specimens across all chemical solutions. The control Specimen, using only water as a solution, exhibited the highest strength, suggesting that this simple approach allows for optimal cement hydration. In contrast, the other solutions likely interfered with hydration, resulting in lower strengths. In Fig. 4b, after 7 days of water immersion, the compressive strength of all Specimens decreased, indicating a susceptibility to moisture. The cement and water solution Specimens, however, retained the highest strength compared to the other mixes, highlighting that cement with a water solution was superior against water penetration. The reduced strength could be due to the leaching of soluble components or changes in pore structures after water immersion. The cement Specimens exhibited a more significant strength reduction, while lime specimens showed lesser changes but less compressive strength in general. Moreover, it should be noted that the lime specimens maintained their volume and dimension before and after water penetration and the cement-based Specimens increased in volume and dimension after water penetration.

A thorough analysis of the results revealed a pronounced impact of 7-day water immersion on the specimens. As depicted in Fig. 4a, the specimen prepared with cement binder and water solution exhibited the highest compressive strength both before and after immersion. Conversely, the prepared bio-composite displayed a softening coefficient of 0.55 when subjected to water, indicating that water is the most effective agent for extracting sugars from wet sawdust. Similar findings were reported by Wai^53^, who concluded that a 24-h water immersion of sawdust led to a significant decrease in water-soluble sugars and tannins.

This suggests that water immersion negatively impacts the structural, integrity of the composite, mainly through wet sawdust swelling and the reduction in bonding between the wood and the matrix. The softening coefficient of 0.55 indicates the magnitude of this strength reduction due to swelling wood sawdust and softening in the water. The decrease in strength could also be due to water penetrating the material, changing the pore structure and weakening the binding between the components. In another study^54^, sawdust was immersed in water and Ca(OH)_2_ solution. This sawdust was subsequently incorporated into sawdust concrete. Higher compressive strengths were obtained for specimens containing sawdust immersed in water compared to those containing sawdust immersed in Ca(OH)_2_ solution after 28 days of hydration. Similarly, in the present study, as illustrated in Fig. 4a, compressive strength values of 5.5 MPa were achieved after immersing sawdust in solutions for 7 days, compared to specimens containing sawdust immersed in other solutions, with a corresponding density of 1300 kg/cm^3^. Additionally, as shown in Fig. 4b, the specimens were immersed, and the results indicated that in this case as well, the specimens with cement binder and water solution exhibited favorable compressive strength, experiencing a reduction of approximately 45% and maintaining the highest strength. Moreover, a 13% increase in density was observed, indicating the superior strength and durability of specimens with cement binder and water solution. Consequently, a strategic decision was made to exclusively utilize wet sawdust treated with water. The improved performance of cement-based materials when compared to lime can be due to better hydration and higher strength characteristics of the binder, or possibly due to slow hardening time and solubility of lime. The reduced strength of the Specimens with Al2(SO_4_)_3_, Ca(OH)_2_, and CaCl_2_ solutions could be attributed to the interaction of these chemicals with the wood components and also the cement matrix, affecting the overall material structure and its mechanical strength.

**Fig. 5:**
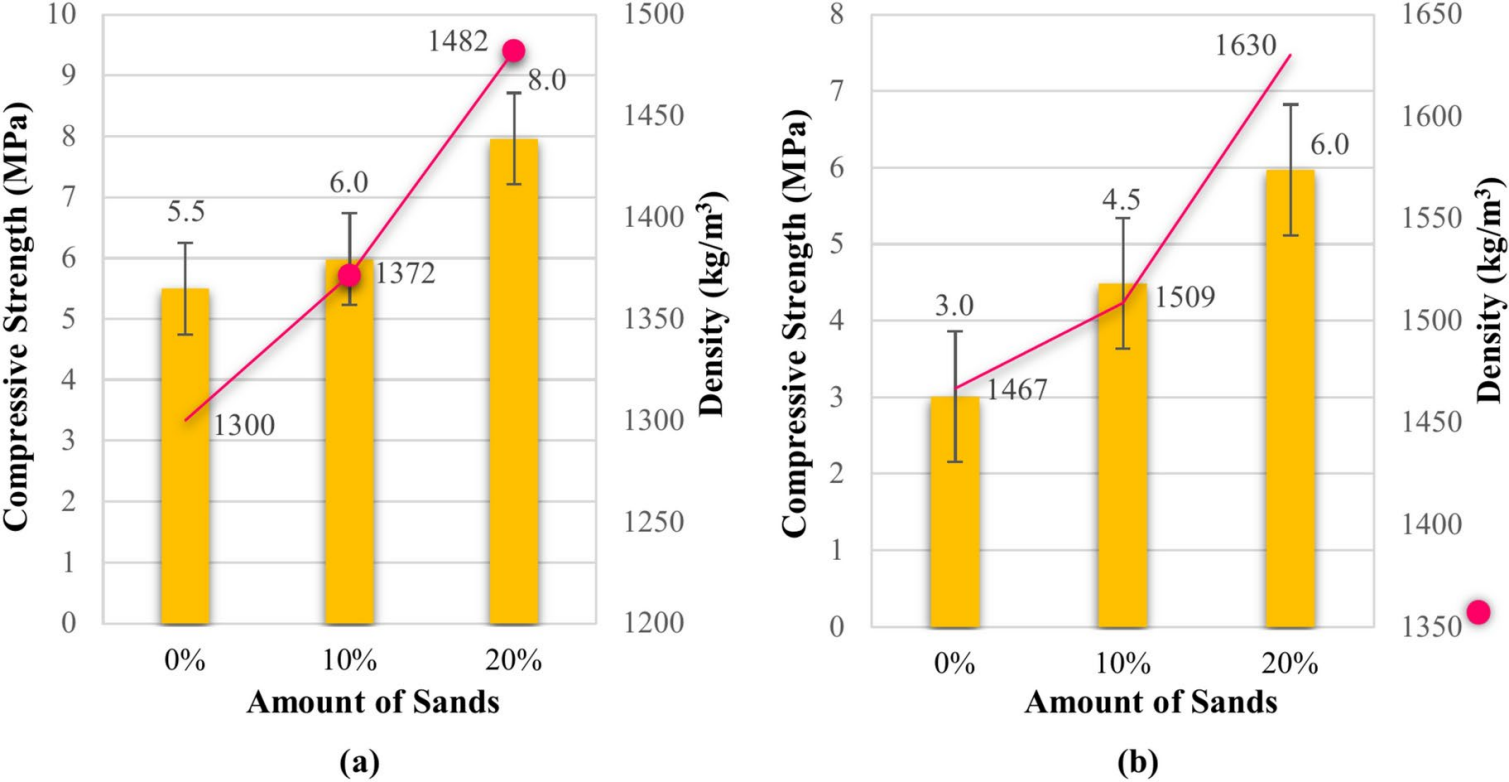
Compressive strength and density of cement-sand bio-composite specimens: (**a**) before water exposure and (**b**) after water exposure.

Building upon this optimized approach, our research subsequently focused on evaluating the impact of incorporating aggregate to enhance the compressive strength of the bio-composite Specimens. To this end, two distinct compositions were meticulously prepared: one containing 10% sand and another with 20% sand, both with a particle size of 0.4 mm, combined with water-treated wet sawdust. These precise experiments aimed to elucidate the role of sand as an additional component in enhancing the strength of wet sawdust-based bio-composites. Figure 5 presents the compressive strength and density values of the bio-composite Specimens with varying sand content. The inclusion of sand, in addition to enhancing compressive strength and density, also serves to improve the bonding and homogeneity of the material. The addition of sand to bio-composites can contribute to improved material bonding and homogeneity. Aggregates can function as mechanical fillers, filling the voids between the organic components, which can lead to increased density and material cohesion. Greater cohesion within the composite structure can improve mechanical properties and reduce the likelihood of failure and cracking. Figure 5a presents the compressive strength and density of the Specimens with varying amounts of sand before submersion and shows that increasing the sand content from 0 to 20% led to a notable increase in both compressive strength and density. This suggests that sand particles act as a filler material, creating a denser and more compact composite structure, which translates into enhanced strength. From Fig. 5b we can see that after 7 days of water immersion, the Specimens displayed a moderate reduction in strength but maintained their density. Again, these Specimens maintained their structural integrity with sand additions, highlighting the positive impact of sand. The increase in density and strength from sand suggests that sand particles are participating in the structure of composite materials.

As shown in Fig. 5a, after a thorough analysis of the results, the impact of adding sand and the optimal mix design were determined. The addition of 20% sand by weight to the bio-composites resulted in an approximate 44% increase in compressive strength and a 14% increase in density. This indicates that the produced bio-composites are lightweight and can be classified as lightweight materials. Similarly to previous series Specimens, and as shown in Fig. 5b, the bio-composites with added sand were also submerged. The results demonstrate that in this condition, they still exhibited satisfactory compressive strength with a reduction of approximately 30% and an 11% increase in density, indicating the favorable resistance and durability effects of adding sand.

**Fig. 6:**
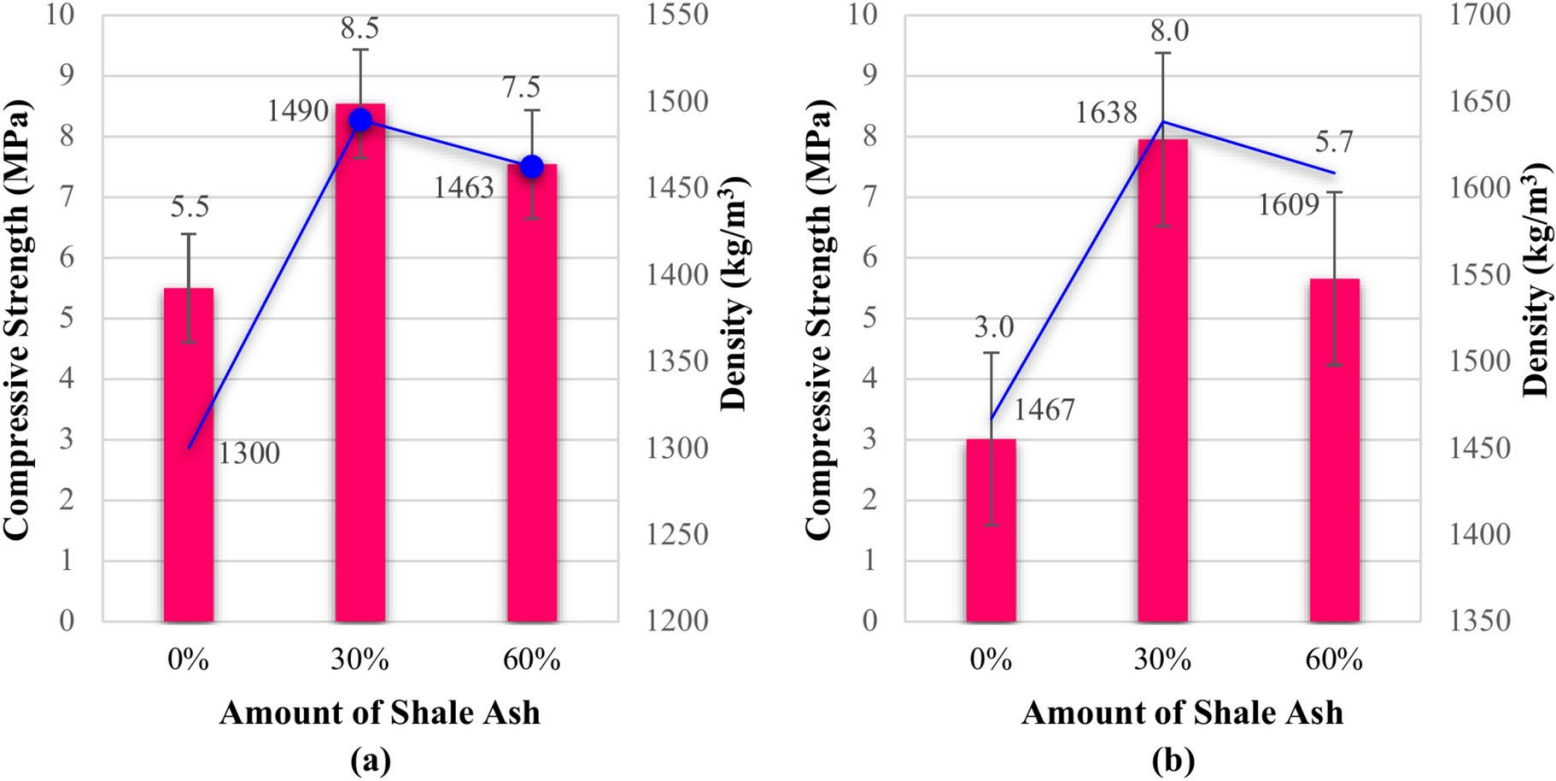
Compressive strength and density of shale ash bio-composites: (**a**) before water exposure; and (**b**) after water exposure.

In a crucial step, a significant decision was made to modify the composition of the Specimens, primarily to reduce the cement content. This strategic adjustment not only decreased the density and weight of the Specimens but also introduced more sustainable materials, aligning with current construction regulations that emphasize environmental compatibility. To implement this change, a specific type of shale ash, which naturally contains sand, was introduced. Consequently, two distinct compositions were carefully prepared: one with 30% cement replacement and the other with 40% cement replacement, both combined with wet wood sawdust treated with water and 20% sand. Figure 6 presents the compressive strength and density values of the bio-composite Specimens with shale ash replacement. Figure 6 shows the effects of partially replacing cement with shale ash in composite materials. Before submersion, Fig. 6a, the Specimen with 30% cement replacement exhibited the greatest compressive strength and density, while 60% replacement resulted in lower properties. This suggests that a 30% substitution of cement with shale ash is an optimal point for enhancing the mechanical properties. As shown in Fig. 6b, after water immersion, these bio-composites also exhibited good water resistance, maintaining their mechanical properties. The decrease in strength was lower than the previous Specimens, highlighting the positive impact of partially replacing cement with shale ash. This improved water resistance might be attributed to the pozzolanic reaction of shale ash, leading to a more durable material.

As demonstrated in Fig. 6a, after a thorough analysis of the results, the optimal replacement percentage of shale ash for cement was determined to achieve the desired mix design. Replacing 30% of the cement by weight with shale ash resulted in a remarkable 55% increase in compressive strength and approximately a 7% increase in density of the produced bio-composites. These findings suggest that substituting shale ash is an efficient approach to reduce cement consumption and produce sustainable bio-composites. Furthermore, similar to the previous series of Specimens, and as shown in Fig. 6b, the bio-composites containing the replaced shale ash were also submerged. The results indicate that, even under submerged conditions, these bio-composites exhibited satisfactory compressive strength, with only a 15% decrease in strength and a 12% increase in density. This demonstrates the effective resistance and durability provided by substituting shale ash for cement, justifying the sustainability of the produced bio-composites from both economic and environmental perspectives.

#### Influence of carbonization process and curing condition

**Fig. 7:**
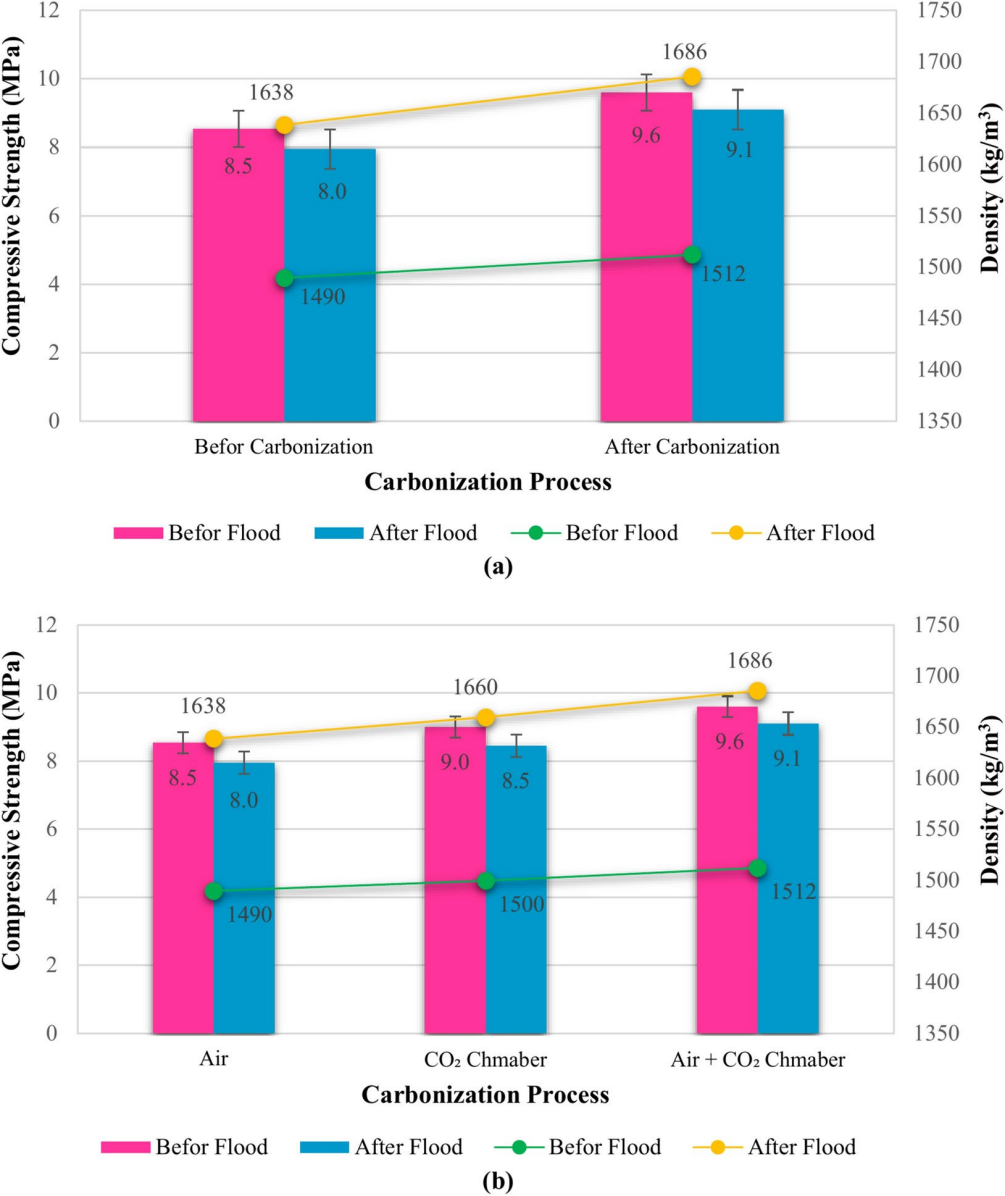
Compressive strength and density of bio-composite Specimens: (**a**) before and after water exposure and carbonization, and (**b**) effect of curing condition.

To achieve an optimal mixture design in terms of compressive strength, density, and water resistance, the potential for improving the properties of bio-composites through the carbonization process was investigated. The results obtained demonstrated that the carbonization process, in addition to increasing strength, also significantly improved durability and overall material properties. Figure 7 presents the compressive strength and density values of the bio-composite Specimens before and after immersion and carbonization. The findings in this section indicate that carbonization, coupled with an appropriate drying/curing regimen, significantly improves the performance of the bio-composites. This is particularly evident in the increased compressive strength and durability under water immersion conditions. This improvement can be attributed to the carbonation of lime components and improved binding within the matrix. Figure 7a shows the effect of carbonization in the specimens, where the specimens that were carbonized in a carbon dioxide chamber demonstrated enhanced mechanical properties. The mechanical properties of the Specimens after water submersion also showed similar results, showing the durability and water resistance of carbonized Specimens. This result shows that the carbonization process improves the mechanical properties of the composite and also makes them more durable under submerged conditions. The improved strength and resistance to water could be due to changes in pore structure, enhanced hydration, and additional binding, which increases resistance to water penetration. The best curing method was to let the Specimens dry before carbonization to have high-quality carbonized bio-composites. As Fig. 7b illustrates, the Specimens cured in a CO_2_ chamber showed intermediate strength, while those cured in ambient air demonstrated the lowest strength. The combined curing method showed the highest compressive strength, which implies that the curing process affects the hydration, carbonation, and overall strength development process.

As depicted in Fig. 7a, a meticulous analysis of the results revealed a substantial enhancement in the compressive strength of the bio-composites upon carbonization. The optimized material composition and curing conditions resulted in an approximate 12% increase in compressive strength and a 2% increase in density compared to the pre-carbonized state. This indicates that carbonization not only improves the mechanical properties but also influences the microstructural and chemical characteristics of the bio-composites. Similarly, when subjected to immersion, carbonized bio-composites exhibited a commendable compressive strength, with only a 15% decrease and a 3% increase in density relative to the pre-carbonized state, highlighting their durability and resistance. Figure 7b further elucidates the significant impact of curing conditions on the compressive strength. Bio-composites cured in a CO_2_ chamber demonstrated intermediate strength, while those cured in ambient air exhibited the lowest strength. The combination of both curing methods yielded the highest compressive strength, a novel finding of this research. Moreover, immediate carbonization post-molding resulted in a decrease in compressive strength. Conversely, allowing the Specimens to dry for a day before carbonization and subsequent curing for 26 days led to a substantial increase in compressive strength. This suggests that carbonization enhances compressive strength in Specimens with reduced initial moisture content. Figure 7a clearly demonstrates that the combined effects of moisture and carbonization lead to a decrease in compressive strength. These results highlight a critical balance: some moisture is necessary to facilitate the carbonization process, but the initial moisture content should be managed to optimize strength. The initial drying before carbonation allows the moisture to evaporate and make more space for the carbonization process, which can enhance the bonding properties and structural integrity of the composite. This means that the initial water content should not be very high but enough to start the chemical reactions.

**Fig. 8:**
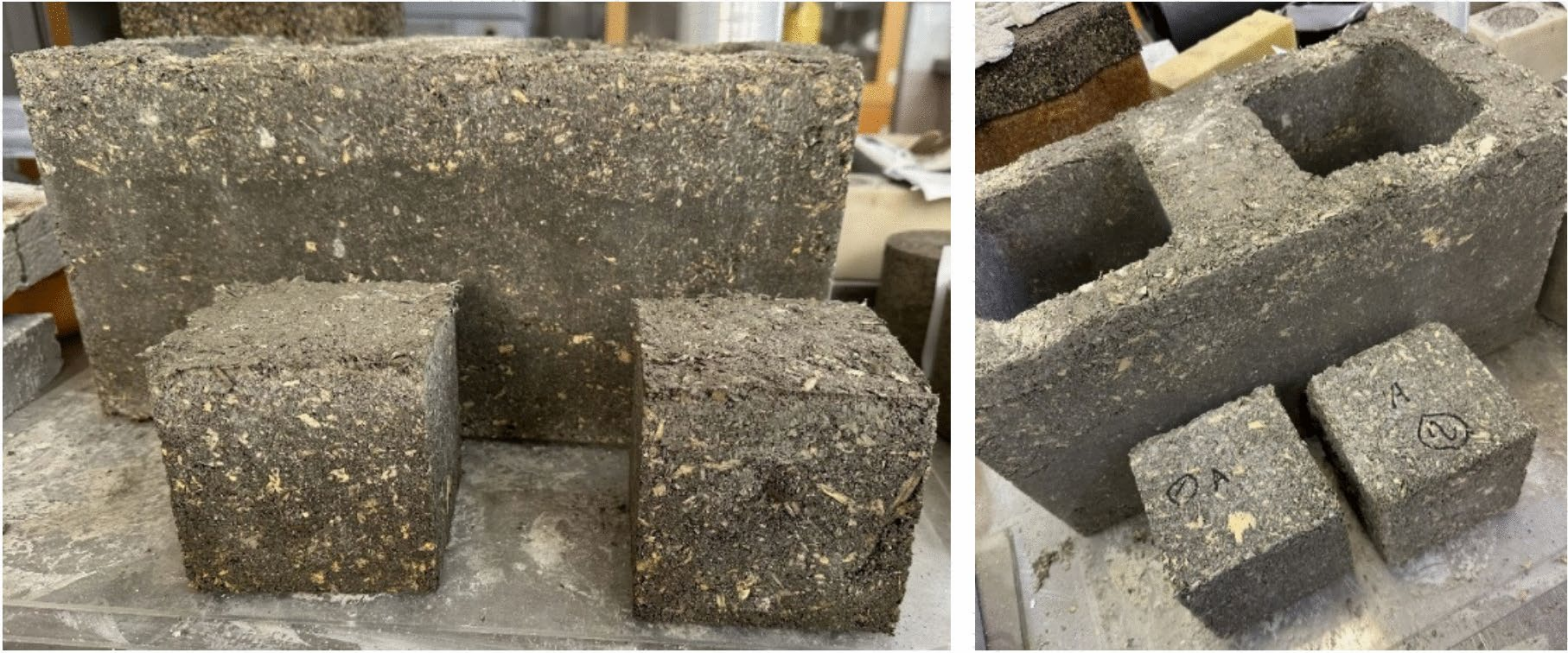
Cubic and block specimens of the same composition.

Subsequent to the meticulous examination of cylindrical specimens, the focus shifted towards cubic specimens and blocks. The primary objective was to validate the feasibility of producing cubic specimens within the predefined parameter range. This critical phase involved assessing the compressive strength of the newly introduced specimens, aiming to precisely determine their mechanical properties. Concurrently, a thorough analysis was conducted to evaluate the practical feasibility of producing blocks using the specified composition. This comprehensive investigation pursued two objectives: not only to confirm the compatibility of the composition with cubic shapes but also to rigorously assess the practical feasibility and structural integrity of the resulting blocks. These results, visually documented in Fig. 8, provide a detailed perspective and significantly contribute to a broader understanding of the potential applications of this material in formal settings. The successful creation of blocks demonstrates the feasibility of the proposed technology and its potential in industrial construction applications. The structural integrity and performance under simulated conditions support its potential use in load-bearing masonry applications. The blocks made in this study show the potential of these bio-composites to be implemented in construction applications for developing more sustainable materials. The good mechanical strength of the bio-composites makes them an ideal material for construction.

Researchers and practitioners can leverage this knowledge to inform sustainable construction practices and enhance structural design. The visual documentation and data unequivocally demonstrate the successful creation of cubes and blocks using the specified composition. This evidence underscores the material’s adaptability and practical applicability, supported by both visual and numerical evidence.

A comprehensive literature review was conducted to identify the most comparable study in this field. A comparative analysis of the literature is presented in Table 2. The two studies represent distinct approaches to incorporating sawdust in construction materials, both driven by the need for more sustainable and cost-effective solutions, but ultimately with very different results. The current research focuses on developing high-performance bio-composites by carbonizing the wood sawdust, which leads to materials with improved mechanical properties compared with traditional concrete, achieving impressive compressive strengths in the about 1–10 MPa range. This involves a relatively complex preparation method including pre-treatment of sawdust in chemical solutions, carefully optimized mixing ratios, and a two-step carbonization/drying process. These rigorous processes result in a high-performing, lightweight material with the potential for a substantial reduction in CO_2_ emissions.

**Table 2:** Comparison of literature review results.

Feature	Current study (carbonized wood bio-composite)	Shantveerayya et al.^55^ (Sawdust replacement in concrete blocks)
Primary focus	Developing & characterizing carbonized wood bio-composites	Evaluating sawdust as a sand replacement in concrete blocks
Sawdust treatment	Soaked in various chemical solutions, then dried and carbonized	No specific chemical treatment reported
Binder type	Cement, lime, cement-shale ash combinations	Cement
Aggregate	Sand, shale ash, various amounts	Sand (replaced by sawdust) and stone aggregate
Curing method	Air-curing, carbonization process, water immersion	Water curing (standard)
Compressive strength	About 1–10 MPa with optimized carbonized formulations	Achieved between 1 and 3 MPa, varying with sawdust ratio
Effect of water exposure	Decreased strength; softening coefficient of 0.55 reported	Decreased strength, more at high sawdust ratios
CO_2_ emission reduction	Significant reduction due to cement replacement and carbonization	Not specifically reported
Density	Lightweight material; 1500–1600 kg/m^3^ with addition of sand, 1400–1500 with shale ash	Reduction in density reported with higher sawdust inclusion
Overall material suitability	Promising for eco-friendly building; improved strength and durability with optimization	Shows promise in reducing construction cost and environmental impact, but with limitations on structural integrity
Microstructure analysis	Showed the presence of valterite, calcite and proper binding between the wood sawdust and the binder material	Not investigated

In contrast, the study by Shantveerayya et al.^55^ aims to replace sand in concrete blocks with untreated sawdust. This research, while simpler and more practical, demonstrates that the sawdust inclusions have a direct and negative relationship with the compressive strength of the concrete blocks. The observed compressive strengths are in the about 1–3 MPa range, significantly lower than the results of the current study. This is primarily due to the higher porosity caused by the use of wood dust with no pretreatment and without proper carbonization process. In addition, no other aggregate is added to the concrete matrix except the wood sawdust. The lower strength in their study highlights that directly replacing sand with sawdust leads to an overall compromise in performance. While their approach has its merits in terms of reducing environmental impact by reusing waste material and reducing the cost of construction, it is not without limitations in structural terms.

The current study’s approach to sawdust treatment, including soaking in various chemical solutions, followed by carbonization is essential to its success. This method effectively alters the properties of the sawdust, enhancing the mechanical binding between the sawdust and the cement matrix while reducing the amounts of the harmful chemicals in the sawdust. In contrast, no such modification or optimization is done in the study by Shantveerayya, where the sawdust is simply mixed with the cement. This difference in treatment significantly impacts the performance of the final material. The current research also optimized the mix ratios for the ingredients as well as the curing parameters to achieve the best mechanical and structural performance of the produced composites, whereas no information has been reported regarding mix ratio optimization or different curing methods in the study by Shantveerayya, which may also contribute to the lower compressive strength. Further, the analysis done in the current research such as microstructural analysis showed better binding between the wood and the binder with the new composite material which contributed to the higher compressive strength and water resistance of the material, and which was not performed in the previous study.

Both studies show a negative impact of water exposure on material strength. However, the current study addresses this issue with techniques such as carbonization and careful material selection, leading to better water resistance with a softening coefficient of 0.55 compared to the material prepared in the study by Shantveerayya. Further, the current study focuses on cement substitution with shale ash, which directly contributed to a significant reduction of carbon footprint during production, while this aspect was not investigated in the study by Shantveerayya, where the focus was only on reducing construction cost by substitution of sand with wood sawdust.

While both studies explore incorporating wood sawdust into construction materials for enhanced sustainability and to deal with industrial waste, they differ significantly in approach and outcomes. The current study, with its sophisticated material processing and carefully optimized designs, resulted in a high-performance bio-composite suitable for a wider range of structural applications with an emphasis on environmental and economic efficiency. On the other hand, the study by Shantveerayya et al.^55^ offers a simpler approach with a lesser impact on performance suitable for low-cost construction. This highlights the fact that the appropriate method and optimization approach should be chosen based on the desired performance of the final material and its application scenario.

### Mineral composition and microstructural

Cement-wood sawdust composites exhibit unique microstructural and mechanical properties, making them promising candidates for construction applications. Research has shown that formulations incorporating low-density, durable wood sawdust can achieve compressive strengths exceeding 30 MPa^12^. Utilizing specific wood species, such as Triplochiton scleroxylon, can enhance mechanical properties, including moisture resistance and modulus of elasticity, making it a suitable option for construction applications that can improve the mechanical properties, particularly the strength of sawdust-based concrete specimens^56^. Another study has demonstrated that the microstructure of cement-wood sawdust composites reveals effective bonding between wood particles and the cement matrix, especially when using magnesium oxysulfate cement (MOSC) which exhibits better compatibility compared to ordinary Portland cement (OPC)^57^. Furthermore, cellulose nanofibers derived from sawdust can enhance the flexural strength of cement-based composites, indicating strong interfacial bonding between the particles^58^. Despite the significant advantages of using sawdust in cement-based composites, challenges such as the chemical incompatibility of certain wood types with cement remain a concern, necessitating careful material selection for optimal performance. Further research in this area is warranted.

**Fig. 9:**
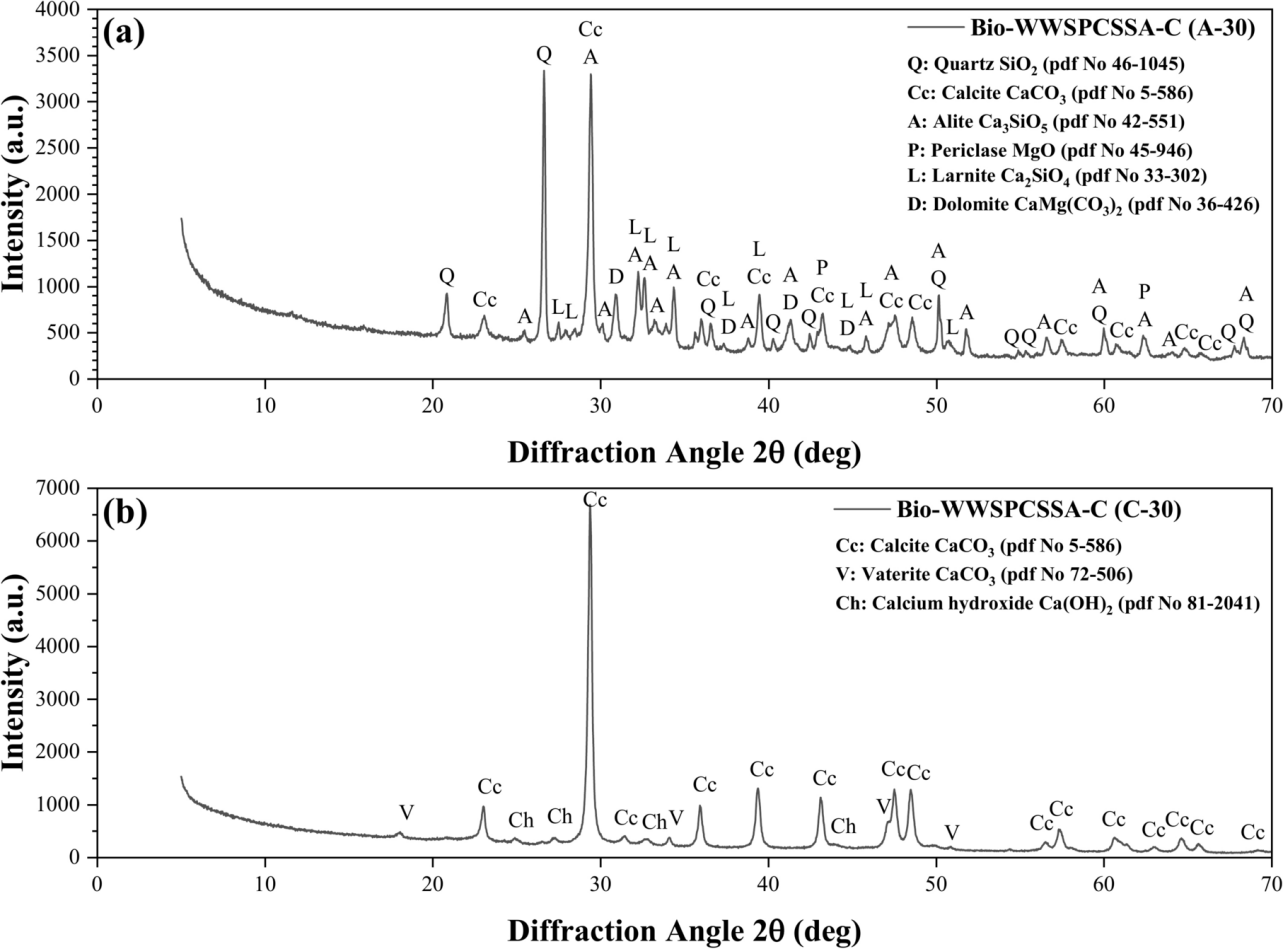
XRD spectra of (**a**) Specimens of composition Bio-WWSPCSSA-C (A-30), primarily composed of cement, and (**b**) Specimens of composition Bio-WWSPCSSA-C (C-30), consisting mainly of lime.

Figure 9 presents the XRD patterns of compositions Bio-WWSPCSSA-C (A-30 and C-30), which are cement and lime binders, respectively, cured in the room after the carbonation process. As evident, due to the consumption of cement in composition Bio-WWSPCSSA-C (A-30), the peaks of compositions Bio-WWSPCSSA-C (A-30 and C-30) are not similar and are significantly different from those Specimens of composition Bio-WWSPCSSA-C (C-30). However, the compressive strength of Specimens with composition Bio-WWSPCSSA-C (A-30) is approximately 92% higher than that of Specimens with composition Bio-WWSPCSSA-C (C-30). The impact of using cement in increasing the strength of the bio-composite Specimens by 92% is a significant finding from both engineering and economic perspectives for the production of low-cost wood-based materials.

**Fig. 10:**
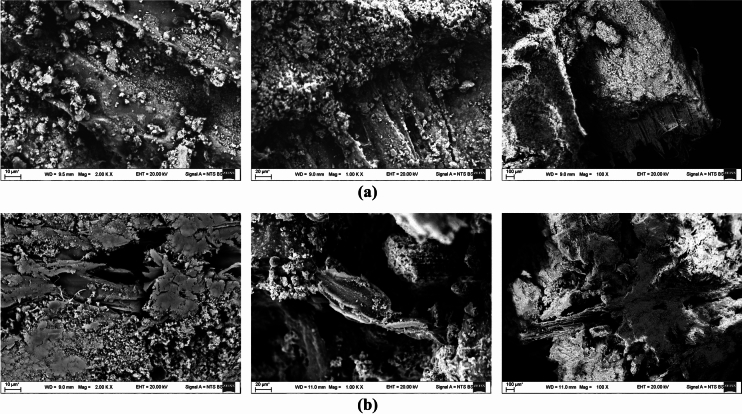
SEM images of bio-composite specimens of compositions (**a**) Bio-WWSPCSSA-C (A-30) and (**b**) Bio-WWSPCSSA-C (C-30) at magnifications of 100x, 1000x, and 2000x.

Figure 10 shows SEM images at magnifications of 100, 1000, and 2000 times for Specimens of compositions Bio-WWSPCSSA-C (A-30 and C-30) made with cement and lime cured in the room after the carbonation process. These images indicate the role of the cement-wood sawdust matrix in binding the cement particles together. Although voids between the particles, resulting from the evaporation of excess physical water, are also clearly visible, it can be easily stated that the structure of the cement-based bio-composite is more continuous and denser, which also contributes to increased strength.

The chemical treatment process is crucial for removing sugars and other materials that could hinder cement hydration. However, the microstructural analysis confirmed that the use of different chemical solutions during the soaking stage had a limited influence on the overall microstructural properties of the produced composites. The water-treated sawdust resulted in the highest strength, indicating that it is the most effective in improving sawdust for cement hydration. This was reinforced by the XRD results, which showed that water treated Specimens had the most crystalline structure compared to Specimens treated with other chemical solutions.

Regarding the presence of valterite and calcite in Specimens of composition Bio-WWSPCSSA-C (A-30 and C-30), it can be noted that when valterite or calcite are incorporated into a cementitious matrix, the microscopic structure of the Specimen changes, which can influence compressive strength. Additionally, chemical reactions between valterite, calcite, and other Specimen components can impact its mechanical properties. Further research is needed to fully understand the complex interplay between these minerals and their impact on the long-term performance of the material.

### CO_2_ emission

The process of producing cement is heavily reliant on energy and involves several stages: the preparation of raw materials, the preparation of fuel, and the final grinding. It has been noted that global cement production has been on an upward trend since the 1990s and is expected to continue to rise until 2050. Cement ranks as the second most used resource after water, with its demand growing yearly due to infrastructure development in both developing and developed countries^59^. CO_2_ emissions in kg per 1kg cement produced for dry and wet cement production process for various fuels and clinker/cement ratios presented in Table 3.

**Table 3:** CO_2_ emissions per unit cement produced (kg CO_2_/kg cement) for dry and wet processes^59^.

Process emissions		Process and fuel-related emissions (CO_2_)							
		Dry process				Wet process			
Clinker/cement ratio	Clinker	Coal	Fuel oil	Natural gas	Waste	Coal	Fuel oil	Natural gas	Waste
55.00%	0.28	0.55	0.50	0.47	0.36	0.67	0.59	0.53	0.36
75.00%	0.38	0.72	0.66	0.61	0.47	0.88	0.77	0.69	0.47
(Portland) 95.00%	0.49	0.89	0.81	0.75	0.57	1.09	0.95	0.90	0.57

To estimate the carbon dioxide emissions based on the biogenic carbon content of the product and the volume, density, and moisture content of the wood, below formula was utilized^60^:

1 $$P_{{CO_{2} }} = \frac{44}{{12}} \times cf \times \frac{{\rho_{a} \times V_{a} }}{{1 + \frac{\omega }{100}}}$$

In this study, the biogenic carbon oxidized as CO_2_ emissions (P_CO2_) from the product system into the atmosphere is quantified, considering factors like energy use at the end-of-life. The carbon fraction (C_f_) of the woody biomass is set at 0.5 by default, representing the oven dry mass. The moisture content (ω) of the product, the density (ρω) of the woody biomass at that moisture content, and the volume (Vw) of the solid wood product at that moisture content are also considered.

With a moisture content of 30%, a wet density of 359 kg/m^3^, a volume of 1 m^3^, and a carbon fraction of 0.5, the absorbed (P_CO2_) for wood sawdust is calculated to be approximately 505 kg. CoMParing CO_2_ emissions from dry and wet cement production processes, the dry process with a clinker/cement ratio of 95% and a coal factor of 0.890 results in 890 kg of CO_2_ per 1000 kg of cement. For the first composition with 1050 kg of cement, this equates to 935 kg of CO_2_, which, after accounting for the (P_CO2_) of the wood sawdust, leaves a net emission of approximately 430 kg. The wet process, with a coal factor of 1.09, results in higher emissions 1090 kg of CO_2_ per 1000 kg of cement, leading to a net emission of approximately 640 kg for the same composition, marking a 48% increase over the dry process. For the final composition with 733 kg of cement, the dry process results in approximately 652 kg of CO_2_ emissions, which, after subtracting the (P_CO2_) of the wood sawdust, leaves a net emission of about 148 kg. The wet process results in approximately 799 kg of CO_2_ emissions, with a net emission of about 294 kg after accounting for the (P_CO2_), showing a 99% increase in emissions compared to the dry process.

The creation of pressed bio-composites from wood waste holds great promise for developing environmentally friendly and sustainable materials^61^. Beyond waste reduction, incorporating wood waste into bio-composite production yields materials with desirable properties, including high strength^62^. However, water’s impact extends beyond biological specimens; it significantly affects structural components within buildings^63^. Prolonged exposure to water can lead to a substantial reduction in compressive strength, up to 55%. This underscores the importance of comprehensive mitigation strategies and heightened awareness regarding the intricate relationship between water and the integrity of both specimens and building materials^64,65^.

## Conclusion

In conclusion, this research demonstrates the feasibility of producing construction materials that are environmentally friendly and durable by combining wood waste with locally available materials such as cement, lime, sand, and shale ash. The outcomes of this work provide valuable insights for further study in the area of sustainable building practices, and emphasizes the importance of optimizing mixing compositions and carbonization techniques to produce bio-composites that are both environmentally and structurally sound. Specifically, this material has the potential to be used as a sustainable alternative for hollow blocks. Based on the findings of this research, several significant conclusions can be drawn:

1. Prolonged water exposure significantly reduces the compressive strength of the materials, as shown by a softening coefficient of 0.55, highlighting the need for strategies to mitigate water damage and a deeper understanding of water-material interactions.
2. Cement with water proved superior to lime as a binder, and adding 20% of 0.4 mm sand to the cement–water mixture significantly improved compressive strength (44%) and density (14%).
3. Replacing some cement with shale ash and adding sand significantly improved the material, resulting in a 55% higher compressive strength and 7% greater density, while also providing cost savings and reducing environmental impact.
4. Carbonization, especially when combined with a specific air drying, CO_2_ chamber conditioning, and ambient curing process, significantly improved bio-composite mechanical properties and durability, both dry and submerged. This process, which included pre-drying before carbonization (19% CO_2_, 65% RH), increased compressive strength by 12% and density by 2%, highlighting the importance of the curing procedure and pre-drying.
5. Optimized bio-composite mixtures, achieved by substituting 30% of Portland cement with shale ash, demonstrated a consistent increase in compressive strength from 0.2 to 9.6 MPa, suitable for lightweight masonry blocks. This substitution not only reduced cement usage and improved sustainability but also enhanced the environmental friendliness of the resulting material.
6. This study achieved higher-strength (1–10 MPa) carbonized wood bio-composites with significant CO_2_ reduction compared to a literature study using sawdust-sand replacement (1–3 MPa), demonstrating the effectiveness of material treatment and optimization for high-performance sustainable materials, although both contribute to sustainable building with different performance levels.
7. XRD analysis revealed that carbonization of bio-composites with Portland cement and lime resulted in the formation of calcite (CaCO_3_), which correlated with increased compressive strength; SEM imaging confirmed the cohesive nature of the wood sawdust and binder paste despite the presence of voids in the bio-composite microstructure.
8. Replacing some cement with shale ash and adding wood sawdust in bio-composite production significantly lowered CO_2_ emissions, with drying and flood processes achieving reductions of approximately 65% and 45%, respectively, demonstrating the potential of bio-composites to reduce the environmental impact of traditional building materials.

## Data Availability

All data generated or analysed during this study are included in this published article.
